# Supplementary material for: The Neurobehavioral Phenotype of School-Aged, Very Prematurely Born Children with No Serious Neurological Sequelae: A Quality of Life Predictor
Source: Children (Basel). 2021 Oct 20;8(11):943. doi: 10.3390/children8110943 (PMC8622308; doi:10.3390/children8110943)
Supplement: Supplementary file 1 [file children-08-00943-s001.zip › children-1418492-supplementary-done.pdf]

children

Table S1: Comparison between children who had all examinations (clinical, cognitive/behavioral, and QoL) and children who had at least one missing data concerning QoL

	Children who had all examinations (N=178)	Children who had at least one missing data concerning QoL (N=124)	*p*
**Perinatal characteristics**			
GA en WA, *mean (SD)*	26.21 (0.90)	26.30 (0.83)	0.375
Missing data, n	*0*	*0*	
Weight in grams, *mean (SD)*	876.80 (177.83)	882.23 (187.87)	0.799
Missing data, n	*0*	*0*	
Male, *n (%)*	86 (48.31)	57 (48.72)	0.946
Missing data, n	*0*	*7*	
SGA, *n (%)*	11 (6.18)	10 (8.06)	0.493
Missing data, n	*0*	*0*	
Multiple pregnancies, *n (%)*	67 (37.64)	36 (29.27)	0.132
Missing data, n	*0*	*1*	
Severe BPD, *n (%)*	91 (51.70)	70 (57.85)	0.296
Missing data, n	*2*	*3*	
Average age of child at the study inclusion, *mean (SD)*	8.46 (±0.75)	8.49 (±0.75)	0.774
Missing data, n	*0*	*0*	
**Parents' educational level, professional activity and socioeconomic status of family**			
Parents without higher education level, *n (%)*	79 (44.63)	51 (43.22)	0.811
Missing data, n	*1*	*6*	
Professional activity of parents, *n (%)*			0.743
Without professional activity	12 (6.78)	10 (8.40)	
Professional activity of one of two parents	58 (32.77)	42 (35.29)	
Professional activity of both parents	107 (60.45)	67 (56.30)	
Missing data, n	*1*	*5*	
Professional activity of mother, *n (%)*	119 (68.00)	75 (64.10)	0.489
Missing data, n	*3*	*7*	
Professional activity of father, *n (%)*	153 (88.95)	101 (90.99)	0.581
Missing data, n	*6*	*13*	
Elevated FAS Score, *n (%)*	107 (60.45)	61 (52.59)	0.183
Missing data, n	*1*	*8*	
**Quality of life[1], *mean (SD)***			
VSP-Ae global index (evaluation by the child)	69.19 (13.57)	67.88 (13.25)	0.423
Missing data, n	*0*	*15*	
VSP-Ap global index (evaluation by the parents)	69.61 (11.17)	71.63 (10.68)	0.219
Missing data, n	*0*	*63*	
Kidscreen global index (evaluation by the child)	72.23 (17.63)	68.79 (16.35)	0.092
Missing data, n	*0*	*6*	
Kidscreen global index (evaluation by the parents)	69.61 (14.83)	69.49 (13.56)	0.946

Missing data, n	*0*	*6*	
**Neurocognitive assesment[2]**			
**WISC-IV[2a], *mean (SD)***			
FSIQ (Full scale index quotient)	91.62 (14.93)	91.36 (16.44)	0.905
Missing data, n	*0*	*52*	
VCI (Verbal comprehension index)	98.56 (15.23)	97.47 (18.42)	0.627
Missing data, n	*0*	*51*	
PRI (Perceptional reasoning index)	92.19 (15.39)	89.20 (15.00)	0.157
Missing data, n	*0*	*49*	
WMI (Working memory index)	90.68 (13.75)	91.59 (15.95)	0.647
Missing data, n	*0*	*50*	
PSI (Processing speed index)	91.98 (13.64)	93.04 (16.38)	0.597
Missing data, n	*0*	*49*	
**NEPSY-2[2b], *mean (SD)***			
Planification Score (Tower)	10.61 (2.63)	10.60 (2.94)	0.974
Missing data, n	*0*	*54*	
Mental flexibility Score	8.13 (2.84)	8.44 (3.26)	0.454
Missing data, n	*0*	*51*	
Auditive attention Score	8.94 (1.64)	8.92 (1.80)	0.939
Missing data, n	*0*	*62*	
Visual attention Score	10.07 (3.32)	10.90 (3.39)	0.074
Missing data, n	*0*	*52*	
**Goodman-SDQ-parents[3], mean (SD)**			
Emotional symptoms	3.48 (2.42)	3.27 (2.52)	0.456
Missing data, n	*0*	*4*	
Behavioral problems	1.93 (1.91)	1.79 (1.82)	0.524
Missing data, n	*0*	*4*	
Hyperactivity/Inattention	4.54 (2.67)	4.53 (2.82)	0.985
Missing data, n	*0*	*4*	
Relationship problems with others	1.84 (1.86)	2.33 (2.06)	0.031*
Missing data, n	*0*	*4*	
Prosocial behaviors	8.83 (1.55)	8.25 (1.83)	0.003*
Missing data, n	*0*	*4*	
Total difficulty scores	11.79 (6.47)	11.93 (6.21)	0.860
Missing data, n	*0*	*4*	
**Anxiety, mean (SD)**			
Spielberg Index (STAIC)[4]	32.73 (7.90)	32.63 (6.84)	0.910
Missing data, n	*1*	*10*	
**Impairment[5]**			
Language delay[5a], *n (%)*	32 (17.98)	20 (27.40)	0.094
Missing data, n	*0*	*51*	
Delay in visuospatial integration[5b], *n (%)*	23 (12.92)	15 (23.08)	0.054
Missing data, n	*0*	*59*	
Attention deficit disorder[5c], *n (%)*	29 (16.29)	8 (12.90)	0.525
Missing data, n	*0*	*62*	

Dysexecutive disorder[5d], *n (%)*	106 (59.55)	39 (54.93)	0.504
Missing data, n	*0*	*53*	
Ideomotor dyspraxia[5e], *n (%)*	13 (7.43)	14 (18.67)	0.009*
Missing data, n	*3*	*49*	

Data are expressed in numbers (%) or mean (SD); SD: standard deviation. *p* = value of the difference between the groups with available data: $p < 0.05$ *: statistically significant difference.

Abbreviations: GA: gestational age; WA: weeks of amenorrhea; SGA: small for gestational age; BPD: bronchopulmonary dysplasia; FAS: Family affluence scale.

Quality of life (Qol)[1]: VSP-Ae and VSP-Ap: Perceived life and perceived health of adolescents, quality of life questionnaires (assessments by both the child and parents respectively). The scores vary between 0 and 100. A high score indicates a better quality of life. Kidscreen-infants and Kidscreen-parents: quality of life questionnaires (evaluation by children and parents respectively). Scores range from 0 to 100. The higher the score, the better the quality of life.

Neurocognitive assessment[2]: WISC-IV[2a]: Weschler Intelligence Scale for Children - 4th edition; NEPSY-2[2b]: NEuroPSYchological assessment, 2nd edition

Goodman-SDQ (Strengths and Difficulties Questionnaire)[3]: score correlated with the Child Behavior Checklist (CBCL) of Achenbach which included 25 items directed towards the parents. The questionnaire reflects a total assessment of the difficulties and defines five subcategories composed of five items each: **1**/Emotional disorder: mean: 0-3, slightly below mean: 4, high: 5-6, very high 7-10; **2**/Behaviors: mean 0-2, slightly below average: 3, high: 4-5, very high 6-10; **3**/Hyperactivity: mean 0-5, slightly below mean: 6-7, high: 4-5, very high 6-10; **4**/Social Relationship disorder: mean: 0-2, slightly below mean 3, high: 4, very high 5-10. A total difficulty score that adds up the first four items. Each increase of one point in the total difficulty score corresponds to an increase in the risk of developing a mental disorder. The categories were proposed in order to assess whether the child's score is in the mean 0-13, slightly below the mean 14-16, high 17-19 or very high 20-40. **5**/"Prosocial" behavior is counted separately and varies in the opposite direction: mean 8-10, slightly below mean: 7, high: 6, very high 0-5.

STAIC[4]: State-Trait Anxiety Inventory for children. Each STAIC Item constitutes of Spielberger an assigned three-point rating scale with a value of 1, 2, and 3. The final score is obtained by adding the sub-scores for each item and ranges from 20 to 60. The normative data of the STAIC, by age and by sex, were established in the United States (Spielberger, 1973) on the basis of two samples of elementary school children: one consisted of 456 girls and 457 boys, in their fourth, fifth and sixth years of primary school; the other of 281 boys and 357 girls for the same years. Boy: Anxiety-Trait 36.7 SD: 6.32 Anxiety-State 31.0 SD 5.71 Girl: Anxiety-Trait: 38.0 SD: 6; 68 anxiety state: 30.7 6.01. Schematically: standard anxiety state: Normal: 20-40, higher 40 high anxiety.

Impairment[5]: A specific cognitive impairment was considered in the GPQol study if at least one of the five neuropsychological disorders (classification of mental illnesses according to Diagnostic and Statistical Manual of Mental Disorders, fourth edition (DSM-IV)) was observed:

A delay in language if the verbal comprehension index (VCI)[5a] <85. A delay in visuospatial integration if the perceptual reasoning index (PRI) <85 and Rey figure ≤ 10th percentile, in copy mode[5b]. An attention deficit if: auditory attention score <8 and/or visual attention score <8 and if the processing speed index (PSI)[5c] <85. Dysexecutive disorder if the working memory index (WMI) <85 and/or planning score <8 and/or the mental flexibility score <8 and/or inhibition score[5d] <10th. Ideomotor dyspraxia: if Touwen assessment test = complex coordination disorder with anomaly of movement planning and (PRI <85 and/or IVT <85)[5e].
